# Retear rates after rotator cuff surgery: a systematic review and meta-analysis

**DOI:** 10.1186/s12891-021-04634-6

**Published:** 2021-08-31

**Authors:** Umile Giuseppe Longo, Arianna Carnevale, Ilaria Piergentili, Alessandra Berton, Vincenzo Candela, Emiliano Schena, Vincenzo Denaro

**Affiliations:** 1 Department of Orthopaedic and Trauma Surgery, Campus Bio-Medico University, Via Álvaro del Portillo, 200, Trigoria, 00128 Rome, Italy; 2 Unit of Measurements and Biomedical Instrumentation, Campus Bio-Medico University, Via Álvaro del Portillo, 21, 00128 Rome, Italy

**Keywords:** Rotator cuff, Rotator cuff tear, Rotator cuff retear, Risk factors, Timing of retear

## Abstract

**Background:**

Rotator cuff retear (RCR) is one of the main postoperative drawbacks. RCR can be considered a multifactorial issue, which causes are related either to biological than biomechanical factors. The aim of this study was to define the incidence of RCR after surgical treatment at different time points and to identify the main factors influencing the postoperative rotator cuff (RC) healing.

**Methods:**

A systematic review and meta-analysis were performed following the PRISMA guidelines. A comprehensive search of the literature was carried out in July 2020, using PubMed and Cochrane Library databases. Only level 1 and 2 clinical evidence studies were included. Studies were included if patients with preoperative repairable full-thickness RC tears were treated surgically, and if studies reported postoperative RCR confirmed by imaging diagnostic. The association between timing of retear and follow-up time points were investigated using an inverse-variance method of pooling data. A subgroup meta-analysis was performed using the DerSimonian and Laird method for the estimation of the between-study variance, i.e., τ^2^. The association between retear rate after surgery and patients’ age, preoperative tear size, fatty infiltration, postoperative rehabilitation protocol, surgical techniques, and RC repairs was determined by expressing the effect measure in terms of odds ratio (OR) with 95% confidence interval (CI). The Mantel-Haenszel method with 95% CIs was used.

**Results:**

Thirty-one articles were included in this study. The percentage of RCR after surgery was 15% at 3 months follow-up, 21% at 3–6 months follow-up, 16% at 6–12 months follow-up, 21% at 12–24 months follow-up, 16% at follow-up longer than 24 months. The main factors influencing RC healing are both patient-related (i.e., age, larger tear size, fatty infiltration) and not patient-related (i.e., postoperative rehabilitation protocol, surgical techniques, and procedures).

**Conclusions:**

Postoperative RC healing is influenced by patient-related and non-patient-related factors. Further high-level clinical studies are needed to provide highly relevant clinical results.

**Supplementary Information:**

The online version contains supplementary material available at 10.1186/s12891-021-04634-6.

## Background

Rotator cuff (RC) tears are one of the leading causes of shoulder pain [1]. RC repair aims to re-attach the injured tendon in its native location. Despite the increase in the number of RC repair [2, 3], rotator cuff retear (RCR) is one of the main postoperative drawbacks.

RCR can be considered a multifactorial issue, which causes are related either to biological than biomechanical factors. The first ones concern patient-related preoperative characteristics, such as age and tear size. RC retear rate increases with patients’ age [4, 5]. Also, the preoperative tear size has been reported as a factor that negatively influences the RC healing after surgery, as the size increases [5–7]. Other factors that have been related to higher healing failure include diabetes, smoking, preoperative fatty infiltration, and muscle atrophy [8–11]. The biomechanical characteristics of the repaired tendons may also be affected by surgical procedures (e.g., arthroscopic, open, mini-open repairs) and fixation techniques (e.g., single-row, double-row, suture bridge, transosseous repairs) [12–14]. Despite the advantages brought by the evolution of surgical techniques, the debate on which implies a lower retear rate after surgery still remains open [14–16]. Timing of retear may be affected by the postoperative rehabilitation protocols, and structural integrity investigation at different time points of follow-up is controversial [1, 17–19]. Some studies showed that retear is most frequent within 3 to 6 months postoperatively [20, 21]. During this postoperative period, no unanimous agreement exists about the best timing and strategy of rehabilitation protocol [22–24].

RCR rate after surgical repair ranges from 11 to 94% [25]. This high variability of RCR raises several concerns about the standards of medical care for RC diseases and adversely affect patients’ expectations. The highest retear rate could be explained by an inadequate selection of patients, or by the inappropriate surgical procedures. The lowest retear rate may suggest both inadequate diagnostic imaging and follow-up time points to quantify more accurately structural integrity of RC after surgical repair. Furthermore, the timing of retears is not well-defined. To identify the most critical moment in which the probability of retears is higher could suggest modification of activities after surgery, or the application of biological solutions to improve RC healing. Moreover, improving the capability to identify patients at higher risk of retears could suggest alternative treatment options and impact positively on clinicians’ decision making.

Therefore, this meta-analysis aimed to define the incidence of RCR after surgical treatment at different time points. Secondly, this investigation aimed to identify the main factors influencing the postoperative RC healing, with emphasis on preoperative patients’ features, surgical procedures, and postoperative rehabilitation protocol.

## Methods

### Search strategy

A systematic review and meta-analysis were performed following the Preferred Reporting Items for Systematic Review and Meta-Analyses (PRISMA) guidelines [26]. A comprehensive search of the literature was performed from inception through July 2020, in PubMed and Cochrane Library databases. The search was conducted separately by two authors (IP, AC). The following search terms were used: (“Rotator Cuff Injuries” OR “Rotator Cuff Tear*” OR “Rotator Cuff Retear*”) AND (“risk factor*” OR age OR “tear size” OR diabetes OR smoking) AND (“timing of retear*” OR “magnetic resonance imaging” OR MRI OR Arthro-MRI OR ultrasound).

### Criteria for including studies

#### Types of studies

Level 1 and 2 clinical evidence studies, as defined by the Oxford Centre for Evidence-Based Medicine, were included [27]. Nonrandomized studies, retrospective studies, case series, systematic reviews, and meta-analysis were not included. Only full-length English-language articles were considered for inclusion.

#### Types of participants

Studies were considered eligible for inclusion if they enrolled patients with repairable full-thickness RC tears, as confirmed preoperatively by imaging diagnostic (e.g., magnetic resonance imaging - MRI, ultrasound - US, computed tomography - CT). Partial RC tears were excluded. No limitations have been placed for inclusion about the preintervention RC tears size, namely small, medium, large, and massive tears have been included.

#### Types of interventions

Studies were included if RC tears were treated surgically, and if they reported postoperative rehabilitation protocols, including immobilization period, passive and active range of motion (ROM), strengthening exercises.

#### Outcome measures

Studies were eligible for inclusion if RCR was reported as an outcome measure, confirmed by postoperative imaging diagnostic. Only full-thickness defects have been counted for the postoperative retear rate. For those studies reporting postoperative tear according to the classification of Sugaya (types I-V) [28], only types IV and V were considered as representing full-thickness tears.

### Data collection and analysis

#### Studies selection process

After duplicates removal, two reviewers (IP, AC) performed the titles and abstracts screening independently. The subsequent screening of the full-texts was carried out separately by the same two reviewers to examine in detail if studies met the inclusion criteria; in case of disagreement, a third reviewer (UGL) made the final decision. The reference list of the included studies was manually screened to retrieve additional studies, not resulting in the first search.

#### Data extraction

Data extraction was performed independently by two authors (IP, AC) using a predetermined checklist. The following data were extracted from studies that met the inclusion criteria: first author and year of publication, study design and level of evidence, randomization groups, basic patients demographic information (i.e., age, gender), postoperative rehabilitation protocol, i.e., immobilization (Yes/No) and correspondent duration (week), beginning of passive ROM (day), active assisted ROM (mean week), full active ROM (mean weeks), strengthening exercises (mean weeks), surgical technique (i.e., arthroscopy, open, mini-open), preoperative tears size according to Cofield classification as small (< 1 cm), medium (1–3 cm), large (3–5 cm), massive (> 5 cm) [29], RC repair (i.e., single-row, double-row, suture bridge, transosseous), diagnostic imaging tools (i.e., MRI, US, CT), number of patients undergoing postoperative diagnostic imaging and follow-up (mean months), number of retears either in each single randomization group than overall and correspondent retear rate, fatty infiltration of cuff muscles before surgery.

#### Risk-of-Bias assessment

All studies that met the inclusion criteria were assessed for risk of bias by two authors independently (AC, VC). In case of disagreement, a third author (UGL) has been summoned to reach the final consensus. The internal validity criteria list proposed by van Tulder et al. has been considered for assessing the risk of bias [30]. The latter has been modified according to the guidelines from Cochrane Handbook for Systematic Reviews of Interventions [31]. The final list included 15 criteria for assessing selection bias, performance bias, attrition bias, detection bias, and reporting bias. The included domains concerned sequence generation, allocation concealment, blinding of participants, selective outcome reporting, incomplete data addressed, personnel, and outcome assessors. This methodological quality assessment is in accordance with the PRISMA protocol [32]. Each criterion was evaluated, assigning a score of 0 for low risk, 1 for uncertain, and 2 for high risk of bias. Thus, the total score range for quality assessment was 0–30: a high score implied a lower quality level, and a low score implied a higher quality level. The quality level of the included studies was evaluated as high if total score ≤ 5, moderate if 5 < total score ≤ 7, and low if total score > 7.

#### Data analysis and statistical methods

The statistical analysis was performed using R software version i368 3.6.1. The retear rate was calculated as the number of patients reporting a not healed tendon after surgery to the total number of patients undergoing RC surgery. Tendons health evaluations refer to imaging examinations performed at the same follow-up time point.

The association between timing of retear and follow-up time points were investigated using an inverse-variance method of pooling data. Pooled retear rate estimates at different time points were performed subdividing follow-up periods into different subgroups, i.e., within 3 months (≤3 months), after 3 months within 6 months (3 < months ≤6), after 6 months within 12 months (6 < months ≤12), after 12 months within 24 months (12 < months ≤24), and after 24 months (months> 24). The subgroups meta-analysis was performed using a random-effect model and the DerSimonian and Laird method for the estimation of the between-study variance, i.e., τ^2^. The I^2^ statistic was applied to define if there was heterogeneity within results. The heterogeneity was interpreted according to the Cochrane Handbook for Systematic Reviews of Interventions guidelines [31]. A random-effects model has been applied to include heterogeneity among studies.

The association between retear rate after surgery and patients’ age, preoperative tear size, fatty infiltration, postoperative rehabilitation protocol, surgical techniques, and RC repairs, was determined by expressing the effect measure in terms of odds ratio (OR) with 95% confidence interval (CI). The OR indicated the ratio of the probability that retear event occurred after RC surgery to the probability that it did not occur. The Mantel-Haenszel method with 95% CIs was used.

## Results

### Search finding

**Fig. 1:**
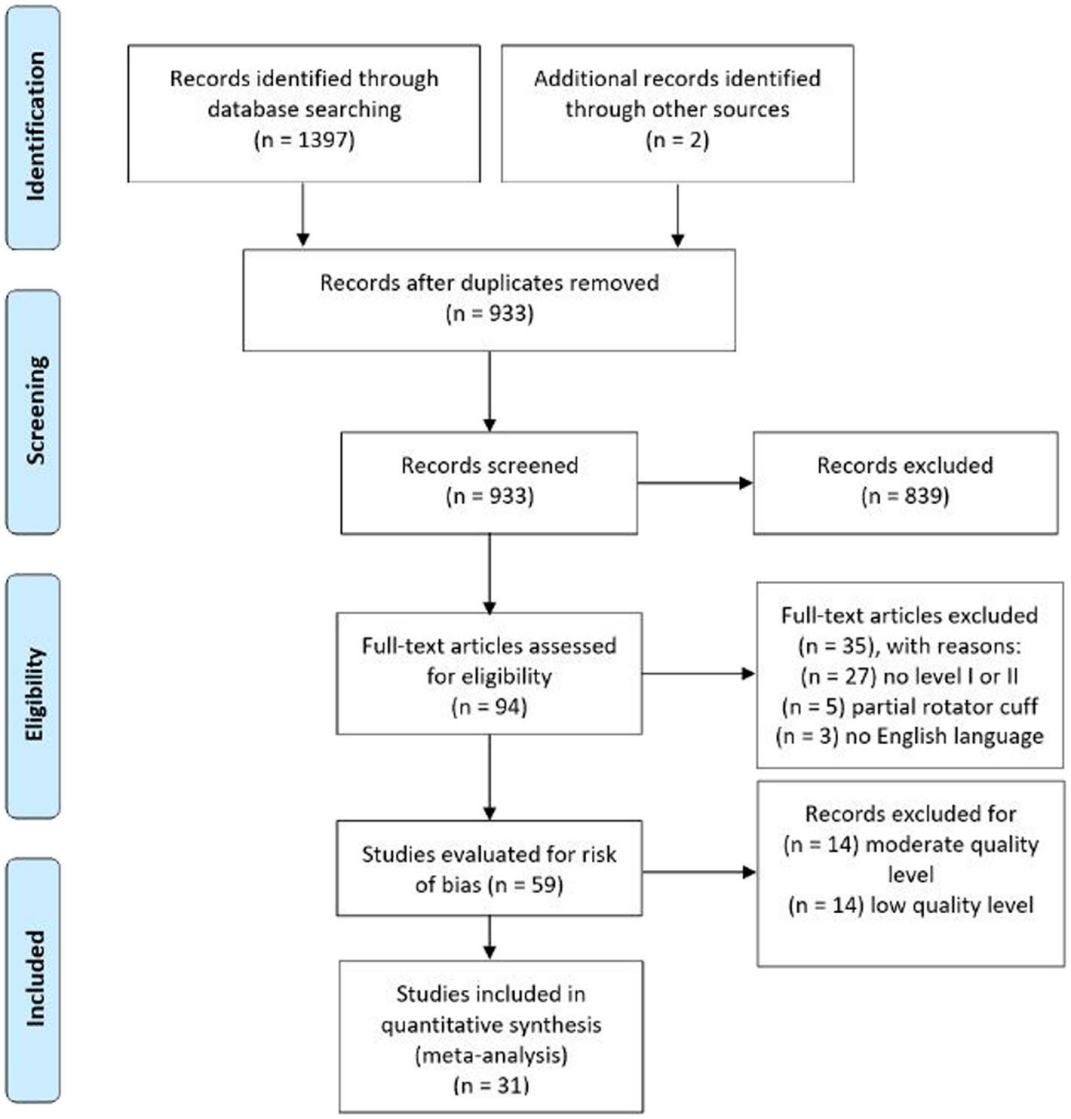
PRISMA flow diagram for studies selection

The results of the literature search, screening, review, and inclusion in quantitative synthesis are reported in Fig. 1. The initial search yielded a total of 1397 articles, with additional 2 articles included after manual reference list screening. After duplicates removal, a total of 933 studies were screened based on title and abstract, of which 839 records were excluded because not relevant for our objective. A total of 94 articles were analyzed in detail, of which 35 records were excluded because they did not satisfy the inclusion criteria. A total of 59 studies were evaluated for methodological quality, 31 of which resulted eligible for meta-analysis [12, 13, 15, 28, 33–87]. None of the studies included the same population. For the complete table, including all extracted data, see Additional file 1 and Additional file 2.

### Risk of bias

The included studies showed an average risk-of-bias score of 5.63 (range, 0–30) (Additional file 3). Of the 59 studies satisfying the inclusion criteria, 31 (52.54%) studies showed a high-quality level, 14 (23.73%) a moderate-quality level, and 14 (23,73%) a low-quality level.

### Timing of retears at different follow-up time points

**Fig. 2:**
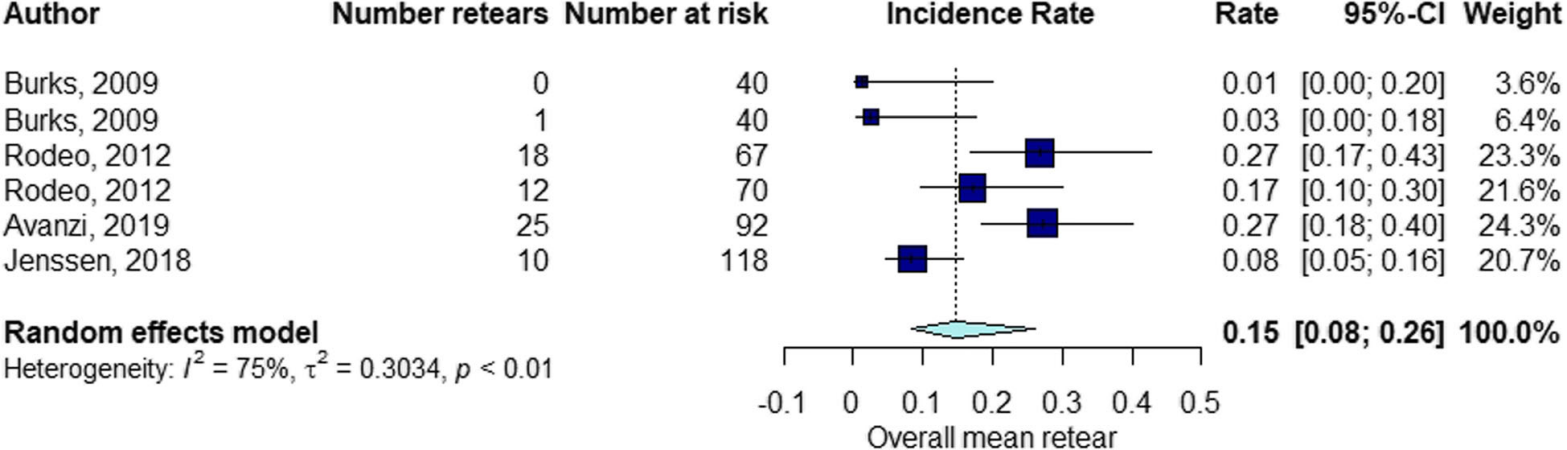
Retear rate within 3 months

**Fig. 3:**
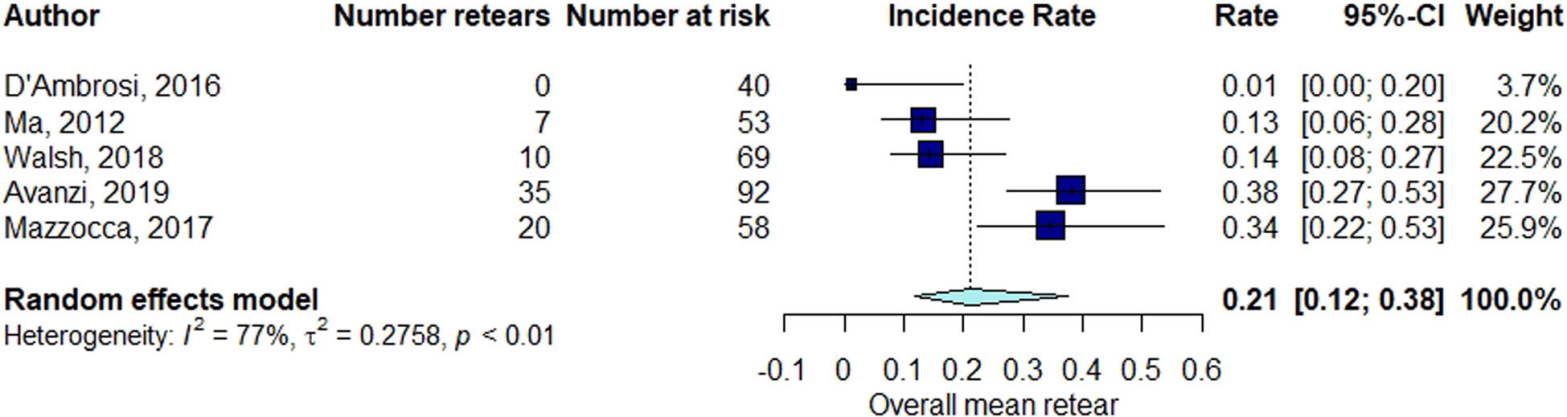
Retear rate within the interval 3 < months ≤6

**Fig. 4:**
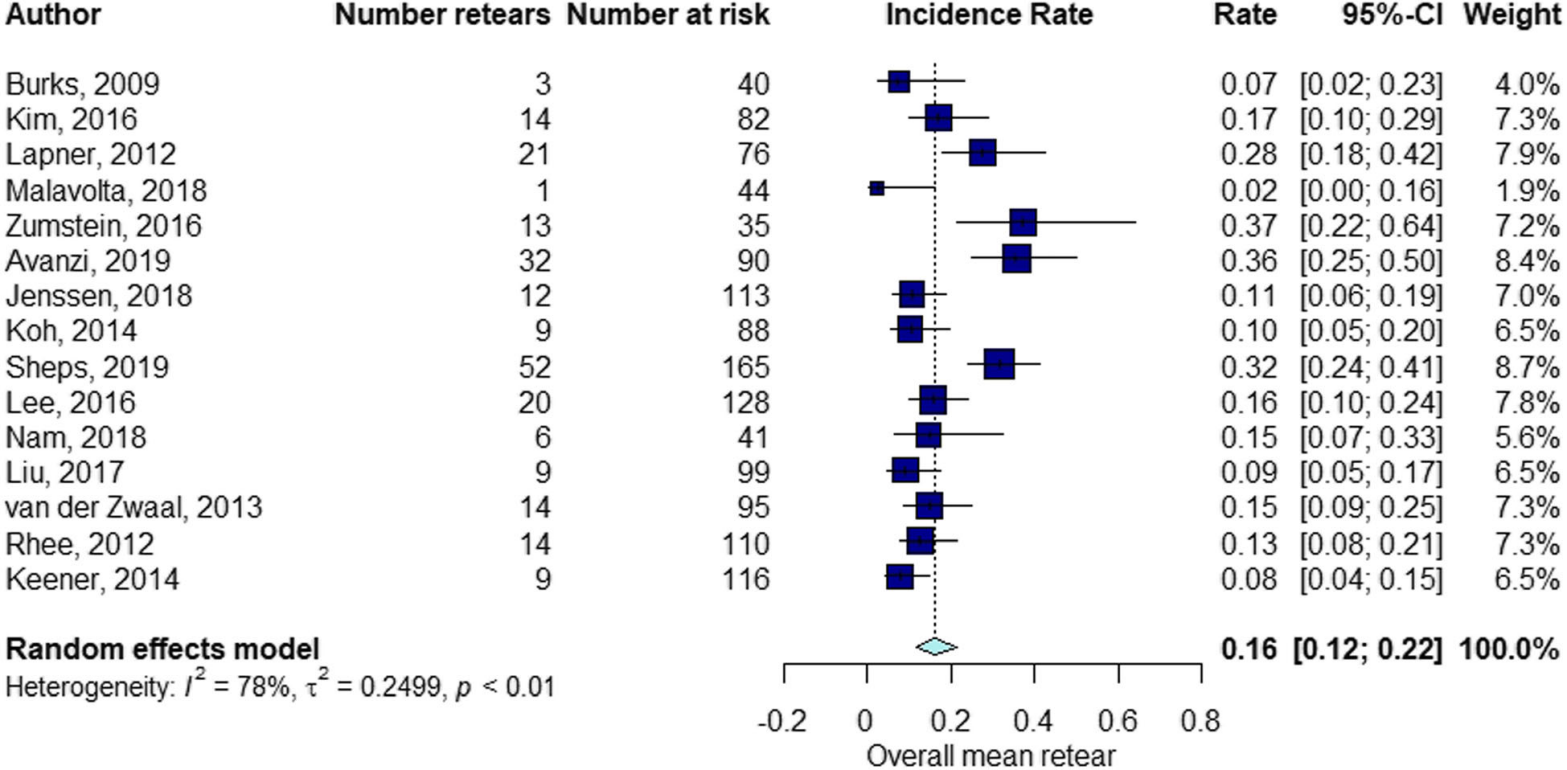
Retear rate within the interval 6 < months ≤12

**Fig. 5:**
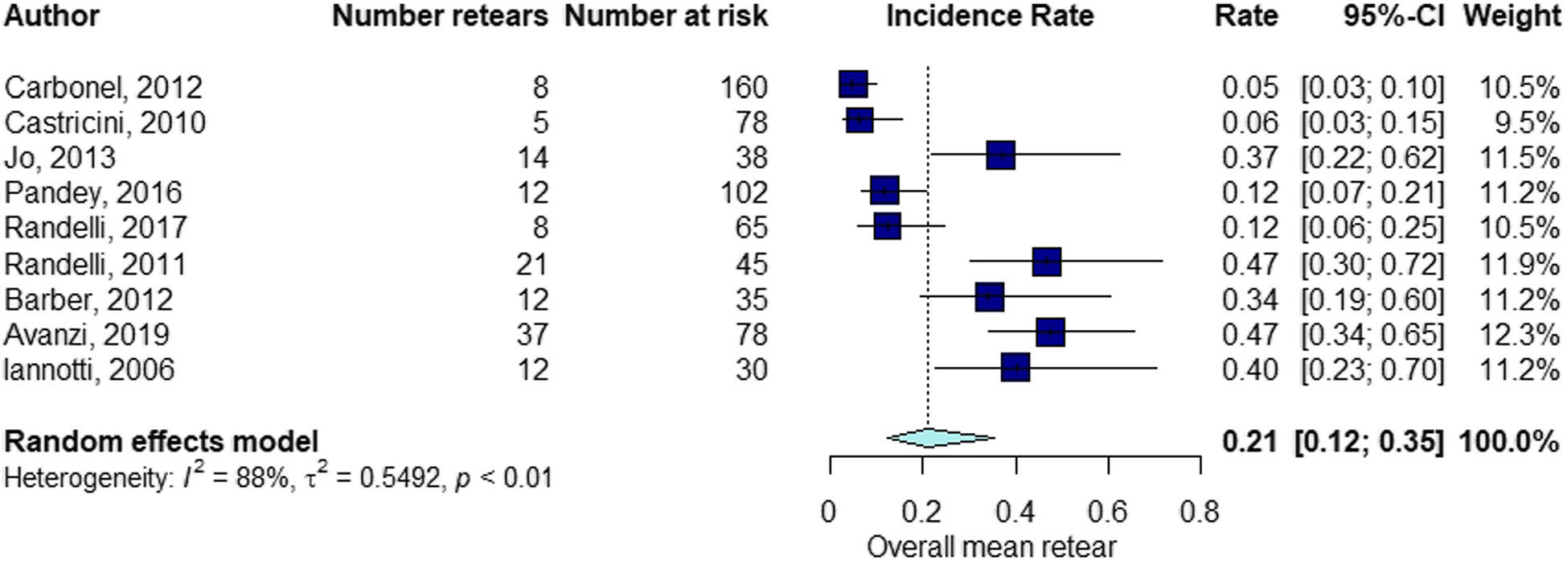
Retear rate within the interval 12 < months ≤24

**Fig. 6:**
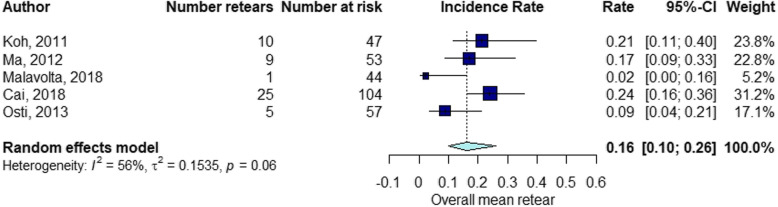
Retear rate over 24 months

The elapsed time between the RC repair and follow-up of structural integrity examination by diagnostic imaging ranged from 1 month to 60 months (mean ± standard deviation [SD], 13.7 ± 11). The percentage of RCR after surgery was 15% at 3 months follow-up (Fig. 2), 21% at 3–6 months follow-up (Fig. 3), 16% at 6–12 months follow-up (Fig. 4), 21% at 12–24 months follow-up (Fig. 5), 16% at follow-up longer than 24 months (Fig. 6).

### Retear rate and patient-related risk factors

#### Age

A total of 31 studies were analysed to examine the relationship between retear rate and patients’ age. The weighted average age of the included studies was 58.2 years (+ 3.7 SD), ranging from 53 to 67 years old. Splitting by different decades of age, studies with a mean age less than equal to 60 years and studies with a mean age over 60 years were compared. The retear rate for patients under 60 years of age was 14.4%. The retear rate for patients over 60 years of age was 24.3%. Older age is associated with higher retear rate (OR, 1.8; 95% CI, 1.5 to 2.3; *P* <  0.0001) (Table 1).

**Table 1:** Comparison of risk factors for retear rate

Risk factors	No. of Studies	Odds ratio (95% CI)	***P***-value
Age	31	1.8 (1.5–2.3)	< 0.0001*
Tear size, A Vs. B^a^	11	0.3 (0.2–0.5)	< 0.0001*
Fatty infiltration (GFDI)	3	0.9 (0.4–1.9)	0.7588
Immobilization period (6 weeks)	22	0.4 (0.1–1.2)	0.0912
Passive range of motion (7 days)	27	0.8 (0.7–1.1)	0.1237
Active assisted ROM (5 weeks)	23	0.5 (0.4–0.7)	< 0.0001*
Full active ROM (8 weeks)	9	2.0 (1.3–3.2)	0.0028*
Strengthening exercises (12 weeks)	23	1.1 (0.8–1.5)	0.4653
Arthroscopic vs. open/mini-open	31	1.0 (0.7–1.7)	0.8524
Single-row vs. double-row	18	1.3 (0.9–1.9)	0.2036
Single-row vs. suture bridge/transosseous	22	0.6 (0.4–0.8)	0.0005*
Double-row vs. suture bridge/transosseous	15	0.5 (0.3–0.7)	0.0001*
PRP vs. No PRP	9	0.6 (0.4–0.9)	0.0179*
Tendon augmentation vs. No augmentation	4	0.2 (0.1–0.4)	< 0.0001*

^a^ A = small, medium, small-to-medium tears, B = large, massive, large-to-massive tears

#### Tear size

A total of 11 studies were analysed to examine the relationship between retear rate and tear size before surgery. Two subgroups were analysed: group A included only small, medium, or small-to-medium tears; group B included only large, massive, or large-to-massive tears. The average retear rate was 12.5% for group A and 37% for group B. Larger tears were associated with higher retear rate (OR, 0.3; 95% CI, 0.2 to 0.5; P <  0.0001) (Table 1).

#### Fatty infiltration

A total of 3 studies were analysed to examine the relationship between the retear rate and presurgical GFDI (global fatty degeneration index). The weighted average of GFDI of the included studies was 1.6 (+ 0.5 SD). Fatty degenerations greater than grade 1.43 correspond to a higher likelihood or retear recurrence [88]. Grade 1.43 of GFDI was used as threshold for discriminating between a high probability of tendon integrity and a high probability of retear. The retear rate was 15.4 and 14.6% for subgroups with GFDI lower than and higher than the threshold, respectively. Results showed no statistically significant difference (OR, 0.9; 95% CI, 0.4 to 1.9; *P* = 0.7588) (Table 1).

### Retear rate and postoperative rehabilitation protocol

#### Immobilization period

A total of 22 studies were analysed to examine the relationship between the retear rate and the immobilization period. According to the rehabilitation protocol of Multicenter Orthopaedic Outcomes Network Shoulder group (the MOON Shoulder Group) [89], the analysis was performed by comparing the retear rate for patients who wore the sling for up to 6 weeks (subgroups 1) to retear rate for patients who were immobilized for more than 6 weeks (subgroups 2). The average retear rate was 17.8 and 8.3% for the subgroups 1 and the subgroup 2, respectively. Results showed no statistically significant difference (OR, 0.4; 95% CI, 0.1 to 1.2; *P* = 0.0912) (Table 1).

#### Passive ROM

A total of 27 studies were analysed to examine the relationship between the retear rate and the beginning of passive ROM. According to the MOON Shoulder Group, in the early group, passive ROM exercises start from 1 day to 1 week after surgery [89]. The analysis was performed by comparing the retear rate for patients who performed early passive ROM (≤7 days after surgery) to retear rate for patients who performed delayed passive ROM (> 7 days after surgery). The average retear rate was 17.5% for the “early subgroup” and 15.6% for the “delayed subgroup”. Results showed no statistically significant difference (OR, 0.8; 95% CI, 0.7 to 1.1; *P* = 0.1237) (Table 1).

#### Active assisted ROM

A total of 23 studies were analysed to examine the relationship between the retear rate and the beginning of active-assisted ROM. The weighted average of weeks of active-assisted ROM of the included studies was 6 (+ 2.6 SD). According to the MOON Shoulder Group, the early active-assisted ROM start before 5 weeks post-surgery and the delayed active-assisted ROM start after 5 weeks post-surgery [89]. Two subgroups were analyzed: the first group and the second group included patients who performed the active-assisted ROM before and after 5 weeks, respectively. The retear rate was 25.6% for the first group, and 14.2% for the second group. Starting active-assisted ROM before 5 weeks corresponds to a higher retear rate (OR, 0.5; 95% CI, 0.4 to 0.7; *P* <  0.0001) (Table 1).

#### Full active ROM

A total of 9 studies were analysed to examine the relationship between the retear rate and the beginning of full active ROM. The weighted average of weeks of full active ROM of the included studies was 8.2 (+ 2.6 SD). According to the MOON Shoulder Group, two subgroups were analysed: the first group and the second group included patients who performed the full active ROM before and after 8 weeks, respectively [89]. The retear rate was 12.1% for the first group, and 21.8% for the second group. Starting full active ROM after 8 weeks corresponds to a higher retear rate (OR, 2; 95% CI, 1.3 to 3.2; *P* = 0.0028) (Table 1).

### Strengthening exercises

A total of 23 studies were analysed to examine the relationship between the retear rate and the beginning of strengthening exercises. The weighted average of weeks of strengthening exercises of the included studies was 11.3 (+ 4 SD). According to the MOON Shoulder Group, strengthening exercises are usually recommended starting at the 12th week after surgery [89]. Two subgroups were analysed: the first group and the second group included patients who performed the strengthening exercises before and after 12 weeks, respectively. The retear rate was 14.5% for the first group, and 15.9% for the second group. Results showed no statistically significant difference (OR, 1.1; 95% CI, 0.8 to 1.5; *P* = 0.4653) (Table 1).

### Retear rate and surgical techniques

#### Arthroscopic versus open/mini-open

A total of 31 studies were analysed to examine the relationship between the retear rate and the performed surgical procedures. Two groups were analysed: the first group included patients who underwent arthroscopic surgery, and the second group included patients who underwent open and/or mini-open surgery. The average retear rate was 17.3% for the first group, and 21.8% for the second group. Results showed no statistically significant difference (OR, 1.0; 95% CI, 0.7 to 1.7; *P* = 0.8524) (Table 1).

#### Single-row versus double-row

A total of 18 studies were analysed to examine the relationship between retear rate and single-row or double-row RCR. The average retear rate was 14.5% for patients who underwent single-row repair, and 12.7% for patients underwent double-row. Results showed no statistically significant difference (OR, 1.3; 95% CI, 0.9 to 1.9; *P* = 0.2036) (Table 1).

#### Single-row versus suture bridge/transosseous

A total of 22 studies were analysed to examine the relationship between retear rate and single-row or suture bridge/transosseous RC repair. The average retear rate was 14.5% for patients who underwent single-row repair, and 23.6% for patients who underwent suture bridge/transosseous RC repair. Suture bridge/transosseous repairs correspond to a higher retear rate than single-row procedure (OR, 0.6; 95% CI, 0.4 to 0.8; *P* = 0.0005) (Table 1).

#### Double-row versus suture bridge/transosseous

A total of 15 studies were analysed to examine the relationship between retear rate and double-row or suture bridge/transosseous RC repair. The average retear rate was 12.7% for patients who underwent double-row repair, and 23.6% for patients who underwent suture bridge/transosseous repair. Suture bridge/transosseous repairs correspond to a higher retear rate than double-row procedure (OR, 0.5; 95% CI, 0.3 to 0.7; *P* = 0.0001) (Table 1).

#### Platelet-rich plasma (PRP)

A total of 9 studies were analysed to examine the relationship between the retear rate and the use of PRP. Two subgroups were identified: the first group and the second group referred to the use or not of PRP, respectively. The average retear rate was 14.5% for the first group, and 23.9% for the second group. The use of PRP corresponds to a lower retear rate (OR, 0.6; 95% CI, 0.4 to 0.9; *P* = 0.0179) (Table 1).

*Tendon augmentation.*

A total of 4 studies were analysed to examine the relationship between retear rate and tendon augmentation for RC repair. Two subgroups were identified: the first group with augmentation and the second group without augmentation. The average retear rate was 21.2% for the first group, and 51.2% for the second group. Tendon augmentation corresponds to a lower retear rate (OR, 0.2; 95% CI, 0.1 to 0.4; *P* <  0.0001) (Table 1).

## Discussion

This study aimed to investigate the retear rate after RC surgery at different time points, also evaluating both patient-related and not patient-related factors. The first ones concern preoperative patients’ characteristics, such as age, tear size, and fatty infiltration. The second ones are related to postoperative rehabilitation protocol, or intraoperative choices of surgical procedures, and RC repairs techniques. The present meta-analysis, including only level 1 and 2 evidence studies, reports data on over 2500 RC repairs.

### RCR at different time points

The results of the present study suggest a difference in retear rate at different follow-up time points corresponding to diagnostic imaging assessments. After surgery, the percentage of RCR was 15% at 3 months follow-up, 21% at 3–6 months follow-up, 16% at 6–12 months follow-up, 21% at 12–24 months follow-up, 16% at follow-up longer than 24 months. These findings would suggest more frequent time points for diagnostic imaging between 3 and 6 months and 12–24 months after RC surgery. Our initial focus was to investigate when the retears occur at different time points in relation to the preoperative tear size. A stratified analysis could not be performed because of the insufficient number of studies reporting the preoperative tear size at different follow-up groups. Despite this limitation, this study could provide significant insights about future studies investigating the retear rate after RC repair. Better knowledge about the timing of retear could be beneficial to define guidelines for surgical procedures and postoperative management.

### Retear rate and patient-related risk factors

The advanced age of patients and larger tear sizes are predictors of RC retear, in agreement with previously published studies [4, 6, 20, 90]. The negative influence of older age on the tendons healing process also depends on other concomitant factors age-related, such as lifestyle, bone mineral density, comorbidities. Some studies report that older age is not an independent predictor of RCR [5]. Larger tear size has been associated with a higher retear rate also in previous systematic reviews [2, 91]. The results of our investigation confirm this association, showing a strong statistical significance (*P* <  0.0001). However, in the attempt to include as many as possible studies in the quantitative analysis, we identified two macro groups. The first group included studies reporting postoperative retear rate for patients with small and/or medium tear size, and the second one included studies enrolling only patients with large and/or massive tear size. For this reason, only 11 studies were analyzed to investigate the relationship between preoperative tear size and retear rate, excluding those studies that enrolled patients independently from the preoperative tear size. Even if 97% of the included studies reported data about patients’ age and preoperative tear size, no one has provided a direct association regarding the number of patients who experienced a retear and their presurgical features. Moreover, the collected information was not enough to perform a stratified analysis, so the definition of the independent effect of both patients’ age and preoperative tear size was not possible.

Current literature reports that RCs with higher muscle fatty infiltration have an increased likelihood of suffering RCR [92]. Our results showed no statistically significant difference (*P* = 0.7588). This deniable statement agrees with the results reported in a recent study [91]. A plausible explanation is that there is no robust scientific evidence. Moreover, studies reporting data on preoperative fatty infiltration did not provide postoperative variations in fatty degeneration and any correlation with RC retear. Although fatty infiltration has been considered as one of the main factors influencing tendons healing after surgery [20, 93], further clinical investigations should be performed to corroborate its impact with exhaustive evidence.

As reported in previous studies, additional patient-related factors that could negatively influence the healing process of the repaired tendons are smoking, diabetes, osteoporosis, hyperlipidemia [5, 6]. In the present work, these risk factors were not analyzed, and further investigations are needed to provide more robust clinical evidence.

### Retear rate and not patient-related risk factors

The biomechanics of the repaired tendons may also be affected by not patient-related factors. In the postoperative period, patients may experience limited functionalities of the affected arm and pain. In this regard, wide debates arise in the definitions of the best rehabilitation programs that should minimize the risk of healing failure and guarantee a successful return to activities of daily living [1, 94]. In the current clinical practice, the postoperative management of patients’ underwent RC repair can be slightly different among studies in terms of time points in which start specific movements and physical exercises. Based on the available literature, the postoperative rehabilitation protocol could be split into four main phases [95]. The first phase refers to the immediate postoperative period until the 6-week during which supervised passive ROM and active-assisted ROM are allowed; in the second phase (weeks 6–12), patients start to execute full-active ROM; in the third phase (months 3–4), stretching and strengthening exercises can be initiated and continued in the fourth phase (months 4–6) to completely restore full and pain-free active ROM and return as normally as possible to sports, activities of daily living and work. The timing of immobilization has been investigated in some recent randomized controlled trials [65, 70, 77]. Longer periods of immobilization may result in shoulder stiffness, which negatively influences the healing process after surgery [81]. Commonly, most patients are asked to wear an abduction pillow for 3 to 6 weeks, during which home postural exercises and assisted ROM during physical therapy are prescribed [65, 84]. The recommended immobilization period may change based on the preoperative tear size. At the same time, the effect of early passive mobilization on the healing rate after RC surgery has been investigated [64, 68, 84]. Compared with the delayed rehabilitation protocol, the early mobilization aims to avoid the likelihood that adhesions would give rise to shoulder stiffness; conversely, delayed rehabilitation protocol seeks to preserve the tendon-to-bone integrity, avoiding retear. One study compared the retear rate in two groups immobilized for four or eight weeks, avoiding any type of passive or active ROM exercises [67]. At a mean of 6.8 months, MRI showed a retear rate of 12.5% for 4-weeks immobilization group and a retear rate of 8.3% for 8-weeks immobilization group. The same study proposed a subgroup analysis, including only patients without preoperative shoulder stiffness. Results showed that at 24-months postoperatively, the 8-week immobilization group had a higher percentage of patients with stiffness [67]. Such findings suggest that the risk of shoulder stiffness might be avoided executing balanced and limited ROM in the first weeks after surgery. Our analysis showed no statistically significant differences for immobilization periods within 6 weeks or longer than 6 weeks postoperatively (OR, 0.4; 95% CI; 0.1 to 1.2; *P* = 0.0912). Some recent meta-analysis investigated the outcomes of early versus delayed rehabilitation [18, 24]. These studies report that early motion protocol corresponds to an increase of ROM after RC repair, but also the risk of retear increases. Based on our findings that larger RC tear size may experience a lower healing rate, we suggest that early motion could be recommended for smaller tear size, while the delayed motion for larger tear size. In the first phase of rehabilitation (within the 5 weeks), active-assisted ROM should be avoided, since a higher retear rate was found for active-assisted ROM starting before 5 weeks (OR, 0.5; 95% CI, 0.4 to 0.7; *P* <  0.0001). In the second phase of rehabilitation (weeks 6–12), full active ROM can be recommended; in particular, our results suggest a higher healing rate if full active ROMs are started before the 8th week (OR, 2; 95% CI, 1.3 to 3.2; *P* = 0.0028). Usually, strengthening exercises are recommended after the 12th week [28, 57, 85], when full active ROM and dynamic shoulder stabilization should be reached [95]. Our results suggest a higher healing rate if strengthening exercises are started before the 12th week, although this result showed no statistically significant difference (OR, 1.1; 95% CI, 0.8 to 1.5; *P* = 0.4653).

During these periods, patients’ compliance with immobilization and prescribed movements should be analyzed [65, 96]. Monitoring patients using wearable technologies could be a plausible alternative if compared to a questionnaire-based investigation [1, 97]. As highlighted previously, tendons healing is strictly associated with factors that surgeons could not handle totally because of dependence from patients’ biological characteristics, as age, tear size, muscles fatty degeneration, and atrophy. Due to the heterogeneity of the included studies and insufficient available data, a stratified analysis for the determination of the independent effect of each factor was not possible to carry out.

In the last decades, arthroscopic RC repair supplanted previous techniques thanks to progress in surgical and technological instrumentations [98]. Faster recovery and better cosmetic results have been the main reasons to prefer the arthroscopic approach. Further studies also supported good clinical outcomes and a low retear rate [99]. The present investigation did not show statistically significant results comparing the retear rate associated with arthroscopic or open and mini-open RC repair (OR, 1; 95% CI, 0.7 to 1.7; *P* = 0.8524).

Our data indicated that double-row techniques yield a lower retear rate than suture bridge/transosseous (OR, 0.5; 95% CI, 0.3 to 0.7; *P* = 0.0001) and single-row RC repair, although there was no statistically significant difference for the latter (OR, 1.3; 95% CI, 0.9 to 1.9; *P* = 0.2036). The present study showed that single-row RC repair is associated with a lower retear rate compared to suture bridge/transosseous RC repair (OR, 0.6; 95% CI, 0.4 to 0.8; *P* = 0.0005).

Double-row RC repair has been described as biomechanically superior compared with single-row [100]. According to our study, numerous systematic reviews and meta-analyses have shown lower postoperative retear rate after double-row repair.

Our findings partially agree with those of Hein et al. that found that double-row had significantly lower retear rate compared with single-row [16]. However, they did not find any significant difference between double-row and suture bridge/transosseous and significantly lower retear rate with suture bridge/transosseous than single-row. Possible reasons for dissimilar results could be patient population, follow-up time, methods for retear diagnosis, and sample size.

As this study focuses on the retear rate based on imaging-classification, no conclusions regarding clinical outcomes as a function of repair technique can be made. Yang et al. demonstrated that postoperative RCR alters clinical outcomes [101]. Future studies should compare differences in the effect based on repair types.

PRP is a promising treatment for some musculoskeletal diseases; however, evidence of its efficacy in the treatment of RC pathologies is still insufficient. Several studies focused on PRP injection for RC tendinopathy, showing benefits over sham injection, no injection, or physiotherapy alone in reducing pain at long-term follow-up [102]. In this review, we analyzed evidence of PRP in arthroscopic repair of RC tears compared with conventional surgery. Lower retear rate was observed with the use of PRP, but several aspects need to be further focused. Many of the included studies specifically looked at the use of platelet-rich fibrin matrix for augmentation (PRFM), while others injected PRP directly into the repair site, including leukocyte-rich PRP (LR-PRP) and leukocyte-poor PRP (LP-PRP). The differences in concentration, content, preparation method and delivery technique do not allow to derive definitive conclusions. Moreover, patients were not stratified based on concomitant factors that can affect the retear rate, such as size, chronicity, atrophy, fatty infiltration, patients’ age, use of tobacco products, diabetes, and other patient-related factors.

Previous studies confirm that PRP has effects on RC structural integrity, promoting tendon healing to the bone, but no effects on clinical outcomes were observed after RC repair. As mentioned above, the present review focused on the retear rate based on imaging-classification, and no conclusions regarding clinical outcomes as a function of repair technique can be made. The efficacy of PRP in arthroscopic repair of RC tears remains under investigation.

The results of this review show that augmented RC repair has a lower retear rate. Structural integrity in postoperative imaging has been documented, but literature is still insufficient.

## Conclusions

Retear rate after RC surgical repair is found to be 15% within 3 months after surgery, 16% at 6–12 months follow-up and at follow-up longer than 24 months, 21% at 3–6 months and 12–24 months follow-up. Advanced patients’ age, larger tear size, and fatty infiltrations are factors influencing the RC healing negatively. Future high-level clinical studies should report data on patients’ condition, postoperative rehabilitation protocol, and surgical techniques in a standardized way to perform a more consistent comparison among studies, and so to provide highly relevant clinical results.

## Supplementary Information

**Additional file 1.** Extracted data, including first author and year of publication, study design and level of evidence, randomization groups, basic patients demographic information (i.e., age, gender), postoperative rehabilitation protocol, i.e., immobilization (Yes/No) and correspondent duration (week), beginning of passive ROM (day), active assisted ROM (mean week), full active ROM (mean weeks), strengthening exercises (mean weeks).

**Additional file 2.** Extracted data, surgical technique (i.e., arthroscopy, open, mini-open), preoperative tears size according to Cofield classification as small (< 1 cm), medium (1–3 cm), large (3–5 cm), massive (> 5 cm), RC repair (i.e., single-row, double-row, suture bridge, transosseous), diagnostic imaging tools (i.e., MRI, US, CT), number of patients undergoing postoperative diagnostic imaging and follow-up (mean months), number of retears either in each single randomization group than overall and correspondent retear rate, fatty infiltration of cuff muscles before surgery.

**Additional file 3.** Risk of bias.

## Data Availability

The datasets used and/or analysed during the current study are available from the corresponding author on reasonable request.

## Abbreviations

RCR: Rotator cuff retear
RC: rotator cuff
OR: odds ratio
CI: confidence interval
PRISMA: Preferred Reporting Items for Systematic Review and Meta-Analyses
ROM: range of motion
MRI: Magnetic Resonance Imaging
US: Ultrasound
CT: Computed Tomography
GFDI: Global Fatty Degeneration Index
PRP: Platelet-rich Plasma
PRFM: platelet-rich fibrin matrix for augmentation
LR-PRP: leukocyte-rich PRP
LP-PRP: leukocyte-poor PRP
