# Supplementary material for: Retear rates after rotator cuff surgery: a systematic review and meta-analysis
Source: BMC Musculoskelet Disord. 2021 Aug 31;22:749. doi: 10.1186/s12891-021-04634-6 (PMC8408924; doi:10.1186/s12891-021-04634-6)
Supplement: Supplementary file 1 — Additional file 1. Extracted data, including first author and year of publication, study design and level of evidence, randomization groups, basic patients demographic information (i.e., age, gender), postoperative rehabilitation protocol, i.e., immobilization (Yes/No) and correspondent duration (week), beginning of passive ROM (day), active assisted ROM (mean week), full active ROM (mean weeks), strengthening exercises (mean weeks). [file 12891_2021_4634_MOESM1_ESM.docx]

**Additional file 1**

| **First**  **author** | **Study**  **design** | **Patients**  **enrolled** | **Group** | **Male** | **Female** | **Age**  **(mean)** | **Age**  **(SD)** | **Passivemotion**  **(day)** | **Imm** | **Time**  **Imm**  **(week)** | **Active assisted ROM**  **(weeks_mean)** | **Full active ROM**  **(weeks_mean)** | **Strengthening exercises**  **(weeks)** |
| --- | --- | --- | --- | --- | --- | --- | --- | --- | --- | --- | --- | --- | --- |
| Burks, 2009 [33] | Randomized controlled trial (I) | 40 | Overall |  |  | 56,5 |  | 7 | Yes |  | 5 | 7 | 11 |
|  |  | 20 | Single-row |  |  | 56 |  |  |  |  |  |  |  |
|  |  | 20 | Double-row |  |  | 57 |  |  |  |  |  |  |  |
| Carbonel, 2012 [34] | Prospective randomized clinical study (I) | 160 | Overall | 68 | 92 | 55,5 |  | 7 | Yes |  | 5 | 7 | 11 |
|  |  | 80 | Single-row | 35 | 45 | 55,79 | 6,3 |  |  |  |  |  |  |
|  |  | 80 | Double-row | 33 | 47 | 55,21 | 5 |  |  |  |  |  |  |
| Castricini, 2010 [35] | Randomized controlled trial (I) | 88 | Overall | 40 | 48 | 55,35 |  | 1 | Yes | 3 | Yes, not specified | Yes, not specified | 6 |
|  |  | 43 | PRP | 17 | 26 | 55,5 |  |  |  |  |  |  |  |
|  |  | 45 | No PRP | 23 | 22 | 55,2 |  |  |  |  |  |  |  |
| D’Ambrosi, 2016 [36] | Randomized controlled trial (I) | 40 | Overall | 19 | 21 | 59,95 |  | 21 | Yes |  | 5 |  | 6 |
|  |  | 20 | PRP | 9 | 11 | 57,9 | 8,7 |  |  |  |  |  |  |
|  |  | 20 | No PRP | 10 | 10 | 62 | 10 |  |  |  |  |  |  |
|  |  | 37 | No PRP | 21 | 16 | 63 | 5,9 |  |  |  |  |  |  |
| Jo, 2013 [44] | Randomized controlled trial (I) | 48 | Overall | 24 | 24 | 63.065 |  | 1 | Yes | 4l - 6m | Yes, not specified | Yes, not specified | 12 |
|  |  | 24 | PRP | 10 | 14 | 64,21 | 6,09 |  |  |  |  |  |  |
|  |  | 24 | No PRP | 14 | 10 | 61,92 | 8,36 |  |  |  |  |  |  |
| Kim, 2016 [45] | Randomized controlled study (II) | 82 | Overall | 27 | 55 | 65,35 |  | 30 | Yes | 4 | Yes, not specified | Yes, not specified | Yes, not specified |
|  |  | 48 | Suture bridge | 16 | 32 | 65,2 |  |  |  |  |  |  |  |
|  |  | 34 | Double-row | 11 | 23 | 65,5 |  |  |  |  |  |  |  |
| Koh, 2011 [28] | Randomized controlled trial (I) | 62 | Overall | 20 | 42 | 61,3 |  | 30 | Yes | 3 | Yes, not specified | Yes, not specified | 12 |
|  |  | 31 | Single-row | 9 | 22 | 61,6 | 8,8 |  |  |  |  |  |  |
|  |  | 31 | Double-row | 11 | 20 | 61,1 | 9,1 |  |  |  |  |  |  |
| Lapner, 2012 [13] | Randomized controlled trial (I) | 90 | Overall | 64 | 26 | 56,8 | 8,1 | 1 | No |  | 6 | 12 | 12 |
|  |  | 48 | Single-row | 35 | 13 | 56 | 8,9 |  |  |  |  |  |  |
|  |  | 42 | Double-row | 29 | 13 | 57,8 | 7 |  |  |  |  |  |  |
| Ma, 2012 [46] | Randomized controlled trial (II) | 53 | Overall | 29 | 24 | 61,2 |  | 1 | Yes | 1 | 7 |  | 9 |
|  |  | 27 | Single-row | 15 | 12 | 60,8 |  |  |  |  |  |  |  |
|  |  | 26 | Double-row | 14 | 12 | 61,6 |  |  |  |  |  |  |  |
| Malavolta, 2018 [47] | Randomized controlled trial (II) | 44 | Overall | 15 | 29 | 54,45 |  | 21 | Yes | 6 | 6 | 6 | 12 |
|  |  | 22 | No PRP | 8 | 14 | 54,2 | 6,8 |  |  |  |  |  |  |
|  |  | 22 | PRP | 7 | 15 | 54,7 | 8,4 |  |  |  |  |  |  |
| Pandey, 2016 [48] | Randomized controlled trial (I) | 102 | Overall | 74 | 28 | 54,45 |  | 30 | Yes | 4 | 7 |  | 12 |
|  |  | 52 | PRP | 38 | 14 | 54,8 | 8,4 |  |  |  |  |  |  |
|  |  | 50 | No PRP | 36 | 14 | 54,1 | 8,3 |  |  |  |  |  |  |
| Randelli, 2017 [49] | Randomized controlled trial (I) | 66 | Overall | 27 | 39 | 54,5 | 6,75 | 29 | Yes | 4 | 9 | 9 | 9 |
|  |  | 31 | Transosseous |  |  |  |  |  |  |  |  |  |  |
|  |  | 35 | Single-row |  |  |  |  |  |  |  |  |  |  |
| Randelli, 2011 [50] | Randomized controlled trial (I) | 53 | Overall | 21 | 32 | 60,5 |  | 10 | Yes | 10 days | 4 |  | 8 |
|  |  | 26 | PRP | 8 | 18 | 61,6 | 8,3 |  |  |  |  |  |  |
|  |  | 27 | No PRP | 13 | 14 | 59,5 | 10,7 |  |  |  |  |  |  |
| Rodeo, 2012 [51] | Randomized controlled trial (II) | 79 | Overall | 44 | 35 | 58.055 |  | 1 | No |  | 6 |  | 12 |
|  |  | 40 | PRFM | 23 | 17 | 58,9 | 9,86 |  |  |  |  |  |  |
|  |  | 39 | No PRFM | 21 | 18 | 57,21 | 9,42 |  |  |  |  |  |  |
| Walsh, 2018 [54] | Randomized controlled trial (II) | 72 | Overall | 41 | 31 | 55,9 |  | 14 | Yes | 2 | 11 | 11 | 7,5 |
|  |  | 44 | No PRPFM | 25 | 19 | 54,9 | 9,3 |  |  |  |  |  |  |
|  |  | 28 | PRPFM | 16 | 12 | 56,9 | 6,7 |  |  |  |  |  |  |
| Zumstein, 2016 [57] | Randomized controlled trial (I) | 35 | Overall | 18 | 17 | 65,5 |  | 21 | Yes | 4 – 6 massive | 5 | 6 | 12 |
|  |  | 17 | L-PRF | 10 | 7 | 65 |  |  |  |  |  |  |  |
|  |  | 18 | No L-PRF | 8 | 10 | 66 |  |  |  |  |  |  |  |
| Barber, 2012 [58] | Lesser-quality randomized controlled trial (II) | 42 | Overall | 31 | 11 | 56 |  |  | Yes | 4 to 6 | 4 | 4 | 12 |
|  |  | 22 | Augmentation | 18 | 4 | 56 |  |  |  |  |  |  |  |
|  |  | 20 | No Augmentation | 13 | 7 | 56 |  |  |  |  |  |  |  |
| Cai, 2018 [59] | Randomized controlled study (II) | 104 | Overall | 56 | 48 | 62,1 |  | 1 | Yes | 1 | 6 |  | 8 |
|  |  | 53 | Control | 32 | 21 | 61,3 | 7,6 |  |  |  |  |  |  |
|  |  | 51 | Study | 24 | 27 | 62,9 | 9,7 |  |  |  |  |  |  |
| Avanzi, 2019 [87] | Randomized Controlled Trial (II) | 92 | Overall | 36 | 56 | 67 |  | Yes_not specified | Yes |  |  |  | Yes, not specified |
|  |  | 46 | Augmentation | 14 | 32 | 68 |  |  |  |  |  |  |  |
|  |  | 46 | No Augmentation | 22 | 24 | 66 |  |  |  |  |  |  |  |
| Iannotti, 2006 [62] | Randomized Controlled Trial (II) | 30 | Overall | 23 | 7 | 57,5 |  | 7 | Yes | 1 | 9 | 9 | 11 |
|  |  | 15 | Control | 12 | 3 | 57 |  |  |  |  |  |  |  |
|  |  | 15 | Augmentation | 11 | 4 | 58 |  |  |  |  |  |  |  |
| Jenssen, 2018 [65] | Prospective Randomized Controlled Non-Inferiority Trial (I) | 118 | Overall | 69 | 49 | 55.5 |  | 1 | Yes | 3 to 6 |  |  |  |
|  |  | 60 | 3w immobilization | 37 | 23 | 56 |  |  |  | 3 | 3 |  | 12 |
|  |  | 58 | 6w immobilization | 32 | 26 | 55 |  |  |  | 6 | 6 |  | 12 |
| Koh, 2014 [67] | Randomized Clinical Trial (I) | 88 | Overall | 44 | 44 | 59.9 |  |  | Yes | 4 to 8 |  |  |  |
|  |  | 40 | 4w immobilization |  |  |  |  | 30 |  | 4 | 5 |  | 11 |
|  |  | 48 | 8w immobilization |  |  |  |  | 60 |  | 8 | 9 |  | 15 |
| Mazzocca, 2017 [69] | Lesser-quality randomized controlled trial (II) | 58 | Overall | 40 | 18 | 54.5 |  |  | Yes | 6 |  |  |  |
|  |  | 27 | Delayed | 19 | 8 | 54 | 7 | 28 |  |  | 5 |  |  |
|  |  | 31 | Early | 21 | 10 | 55 | 8 | 2.5 |  |  | 1 |  |  |
| Sheps, 2019 [70] | High-quality randomized controlled trial (I) | 206 | Overall | 131 | 75 | 55.85 |  | 1 | Yes, No | 6 |  |  |  |
|  |  | 103 | Early Mobilization | 65 | 38 | 55.5 | 8.3 |  | Optional |  |  |  |  |
|  |  | 103 | Standard Rehabilitation | 66 | 37 | 56.2 | 10.1 |  | Yes |  |  |  |  |
| Lee, 2016 [71] | Randomized Controlled Trial (I) | 128 | Overall | 29 | 99 | 62.9 |  | 30 | Yes | 4 |  |  |  |
|  |  | 56 | LHBT tenotomy | 11 | 45 | 62.8 |  |  |  |  |  |  |  |
|  |  | 72 | LHBT tenodesis | 18 | 54 | 62.9 |  |  |  |  |  |  |  |
| Nam, 2018 [74] | Randomized Controlled Trial (I) | 71 | Overall | 37 | 34 | 60.2 |  |  | Yes | 5 |  |  |  |
|  |  | 36 | Limited Bursectomy | 17 | 19 | 59.7 | 6.1 |  |  |  |  |  |  |
|  |  | 35 | Extensive Bursectomy | 20 | 15 | 60.7 | 5.4 |  |  |  |  |  |  |
| Osti, 2013 [76] | Randomized Controlled Study | 57 | Overall | 30 | 27 | 60.5 |  | 15 | Yes | 4 | 6 |  | 12 |
|  |  | 28 | Repair and microfractures | 16 | 12 | 61.2 |  |  |  |  |  |  |  |
|  |  | 29 | Repair only | 14 | 15 | 59.8 |  |  |  |  |  |  |  |
| Liu, 2017 [15] | randomized clinical trial | 99 | Overall | 49 | 50 | 53 |  | 6 | Yes |  | 6 |  | 12 |
|  |  | 50 | Arthroscopic | 25 | 25 | 53.5 | 4.3 |  |  |  |  |  |  |
|  |  | 49 | Mini open | 24 | 25 | 52.5 | 5 |  |  |  |  |  |  |
| van der Zwaal, 2013 [79] | randomized controlled trial (II) | 95 | Overall | 57 | 38 | 57.5 |  | No | No |  | 1 |  | Yes_not specified |
|  |  | 47 | Arthroscopic | 29 | 18 | 57.2 | 8 |  |  |  |  |  |  |
|  |  | 48 | Mini open | 28 | 20 | 57.8 | 7.9 |  |  |  |  |  |  |
| Rhee, 2012 [80] | Cohort study (II) | 110 | Overall | 60 | 50 | 59.3 |  | 1 | No |  | 6 |  | Yes_not specified |
|  |  | 51 | Knotless | 30 | 21 | 61 |  |  |  |  |  |  |  |
|  |  | 59 | Knot-Tying | 30 | 29 | 57.6 |  |  |  |  |  |  |  |
| Keener, 2014 [81] | Prospective Randomized Trial (I) | 124 | Overall |  |  | 55 |  | 7 to 42 | Yes | 6 |  |  |  |
|  |  | 65 | Standard Rehabilitation |  |  |  |  | 7 |  |  | 6 |  | 13 |
|  |  | 59 | Immobilization |  |  |  |  | 42 |  |  | 13 |  | 28 |
