# Supplementary material for: Retear rates after rotator cuff surgery: a systematic review and meta-analysis
Source: BMC Musculoskelet Disord. 2021 Aug 31;22:749. doi: 10.1186/s12891-021-04634-6 (PMC8408924; doi:10.1186/s12891-021-04634-6)
Supplement: Supplementary file 2 — Additional file 2. Extracted data, surgical technique (i.e., arthroscopy, open, mini-open), preoperative tears size according to Cofield classification as small (< 1 cm), medium (1–3 cm), large (3–5 cm), massive (> 5 cm), RC repair (i.e., single-row, double-row, suture bridge, transosseous), diagnostic imaging tools (i.e., MRI, US, CT), number of patients undergoing postoperative diagnostic imaging and follow-up (mean months), number of retears either in each single randomization group than overall and correspondent retear rate, fatty infiltration of cuff muscles before surgery. [file 12891_2021_4634_MOESM2_ESM.docx]

**Additional file 2**

| **First**  **author** | **Group** | **Surgical technique** | **Tear size** | **RC repair** | **Imaging** | **Imaging (n)** | **Time imaging (months)** | **Retears number** | **Retears rate** | **Preoperative muscle fatty infiltration** |
| --- | --- | --- | --- | --- | --- | --- | --- | --- | --- | --- |
| Burks, 2009 [33] | Overall | A | M, L | SR, DR | MRI | 40 | 1,5 | 1 | 0,025 |  |
|  |  |  |  |  |  |  | 3 | 0 | 0 |  |
|  |  |  |  |  |  |  | 12 | 3 | 0,075 |  |
|  | Single-row |  |  | SR |  | 20 | 1,5 | 0 | 0 |  |
|  |  |  |  |  |  |  | 3 | 0 | 0 |  |
|  |  |  |  |  |  |  | 12 | 2 | 0,1 |  |
|  | Double-row |  |  | DR |  | 20 | 1,5 | 1 | 0,05 |  |
|  |  |  |  |  |  |  | 3 | 0 | 0 |  |
|  |  |  |  |  |  |  | 12 | 1 | 0,05 |  |
| Carbonel, 2012 [34] | Overall | A | M, L | SR, DR | MRI | 160 | 24 | 8 | 0,05 |  |
|  | Single-row |  |  | SR |  | 80 | 24 | 5 | 0,0625 |  |
|  | Double-row |  |  | DR |  | 80 | 24 | 3 | 0,0375 |  |
| Castricini, 2010 [35] | Overall | A | S, M | DR | MRI | 78 | 20,2 | 5 | 0,064 |  |
|  | PRP |  |  |  |  | 40 | 20,2 | 1 | 0,025 |  |
|  | No PRP |  |  |  |  | 38 | 20,2 | 4 | 0,105 |  |
| D'Ambrosi, 2016 [36] | Overall | A | M, L | SR | US | 40 | 6 | 0 | 0 |  |
|  | PRP |  |  |  |  | 20 | 6 | 0 | 0 |  |
|  | No PRP |  |  |  |  | 20 | 6 | 0 | 0 |  |
| Jo, 2013 [44] | Overall | A | L, MS | SB | MRI, CTA | 38 | 12,24 | 14 | 0,37 | ^a^ SSP (0_0, 1_3, 2_18,3_12,4_15) |
|  |  |  |  |  |  |  |  |  |  | ISP (0_1, 1_23, 2_15,3_1,4_8) |
|  |  |  |  |  |  |  |  |  |  | SSC (0_2, 1_28, 2_13,3_2,4_3) |
|  | PRP |  |  |  |  | 20 | 13,38 | 4 | 0,2 | SSP (0_0, 1_3, 2_10,3_3,4_8) |
|  |  |  |  |  |  |  |  |  |  | ISP (0_1, 1_14, 2_6,3_0,4_3) |
|  |  |  |  |  |  |  |  |  |  | SSC (0_2, 1_12, 2_9,3_0,4_1) |
|  | No PRP |  |  |  |  | 18 | 11,1 | 10 | 0,56 | SSP (0_0, 1_0, 2_8,3_9,4_7) |
|  |  |  |  |  |  |  |  |  |  | ISP (0_0, 1_9, 2_9,3_1,4_5) |
|  |  |  |  |  |  |  |  |  |  | SSC (0_0, 1_16, 2_4, 3_2,4_2) |
| Kim, 2016 [45] | Overall | A | M, L | SB, DR | MRI | 82 | 12 | 14 | 0,17 | 0_5,1_31,2_37,3_7,4_2 |
|  | Suture bridge |  |  |  |  | 48 | 12 | 8 | 0,17 | 0_3,1_18,2_22,3_4,4_1 |
|  | Double-row |  |  |  |  | 34 | 12 | 6 | 0,18 | 0_2,1_13,2_15,3_3,4_1 |
| Koh, 2011 [28] | Overall | A | M, L | SR, DR | MRI | 47 | 27,5 | 10 | 0,213 | ^b^GFDI_1.2 |
|  | Single-row |  |  |  |  | 24 | 27,4 | 4 | 0,167 | GFDI_1.2 |
|  | Double-row |  |  |  |  | 23 | 27,6 | 6 | 0,261 | GFDI_1.2 |
| Lapner, 2012 [13] | Overall | A | S, M, L, MS | SR, DR | MRI, US | 76 | 12 | 21 | 0,28 |  |
|  | Single-row |  |  |  |  | 39 | 12 | 13 | 0,33 |  |
|  | Double-row |  |  |  |  | 37 | 12 | 8 | 0,22 |  |
| Ma, 2012 [46] | Overall | A | S, M, L, MS | SR, DR | MRI, US | 53 | 6 | 7 | 0,132 |  |
|  |  |  |  |  |  | 53 | 33 | 9 | 0,17 |  |
|  | Single-row |  |  |  |  | 27 | 6 | 4 | 0,148 |  |
|  |  |  |  |  |  | 27 | 33 | 6 | 0,222 |  |
|  | Double-row |  |  |  |  | 26 | 6 | 3 | 0,115 |  |
|  |  |  |  |  |  | 26 | 33 | 3 | 0,115 |  |
| Malavolta, 2018 [47] | Overall | A | S, M | SR | MRI | 44 | 12 | 1 | 0,023 |  |
|  |  |  |  |  |  | 44 | 60 | 1 | 0,023 |  |
|  | No PRP |  |  |  |  | 22 | 12 | 1 | 0,045 |  |
|  |  |  |  |  |  | 22 | 60 | 1 | 0,045 |  |
|  | PRP |  |  |  |  | 22 | 12 | 0 | 0 |  |
|  |  |  |  |  |  | 22 | 60 | 0 | 0 |  |
| Pandey, 2016 [48] | Overall | A | M, L | SR | US | 102 | 24 | 12 | 0,118 |  |
|  | PRP |  |  |  |  | 52 | 24 | 2 | 0,038 |  |
|  | No PRP |  |  |  |  | 50 | 24 | 10 | 0,2 |  |
| Randelli, 2017 [49] | Overall | A | S, M, L | SR, TR | MRI | 65 | 15 | 8 | 0,123 |  |
|  | Transosseous |  |  |  |  | 30 | 15 | 4 | 0,133 |  |
|  | Single-row |  |  |  |  | 35 | 15 | 4 | 0,114 |  |
| Randelli, 2011 [50] | Overall | A | S, M, L, MS | SR | MRI | 45 | 23 | 21 | 0,467 |  |
|  | PRP |  |  |  |  | 22 | 21 | 9 | 0,409 |  |
|  | No PRP |  |  |  |  | 23 | 25 | 12 | 0,522 |  |
| Rodeo, 2012 [51] | Overall | A | S, M, L | SR, DR, SB | US | 70 | 1,5 | 12 | 0,171 |  |
|  |  |  |  |  |  | 67 | 3 | 18 | 0,269 |  |
|  | PRFM |  |  |  |  | 34 | 1,5 | 6 | 0,176 |  |
|  |  |  |  |  |  | 36 | 3 | 12 | 0,333 |  |
|  | No PRFM |  |  |  |  | 36 | 1,5 | 6 | 0,167 |  |
|  |  |  |  |  |  | 31 | 3 | 6 | 0,194 |  |
| Walsh, 2018 [54] | Overall | A | M, L | DR | MRI | 69 | 6 | 10 | 0,145 |  |
|  | No PRPFM |  |  |  |  | 42 | 6 | 8 | 0,19 |  |
|  | PRPFM |  |  |  |  | 27 | 6 | 2 | 0,074 |  |
| Zumstein, 2016 [57] | Overall | A | S, M, L, MS | SB | MRI | 35 | 12 | 13 | 0,371 | ^a^ SSP (0_2, 1_19, 2_14, 3_0, 4_0)  ISP (0_14, 1_16, 2_5, 3_0, 4_0) |
|  | L-PRF |  |  |  |  | 17 | 12 | 6 | 0,353 | SSP (0_1,1_8, 2_8,3_0,4_0)  ISP (0_7,1_8,2_2, 3_0, 4_0) |
|  | No L-PRF |  |  |  |  | 18 | 12 | 7 | 0,389 | SSP (0_1, 1_11, 2_6, 3_0, 4_0)  ISP (0_7, 1_8, 2_3, 3_0, 4_0) |
| Barber, 2012 [58] | Overall | A | L, MS | SR | MRI | 35 | 14,5 | 12 | 0,34 |  |
|  | Augmentation |  |  |  |  | 20 | 14,5 | 3 | 0,15 |  |
|  | No Augmentation |  |  |  |  | 15 | 14,5 | 9 | 0,6 |  |
| Cai, 2018 [59] | Overall | A | M, L | SB | MRI | 104 | 28,2 | 25 | 0,24 |  |
|  | Control |  |  |  |  | 53 | 28,2 | 18 | 0,34 |  |
|  | Study |  |  |  |  | 51 | 28,2 | 7 | 0,137 |  |
| Avanzi, 2019 [87] | Overall | A | S, M | SR, DR | MRI | 92 | 1 | 25 | 0,272 |  |
|  |  |  |  |  |  | 92 | 6 | 35 | 0,38 |  |
|  |  |  |  |  |  | 90 | 12 | 32 | 0,356 |  |
|  |  |  |  |  |  | 78 | 24 | 37 | 0,474 |  |
|  | Augmentation |  |  |  |  | 46 | 1 | 3 | 0,065 |  |
|  |  |  |  |  |  | 46 | 6 | 8 | 0,174 |  |
|  |  |  |  |  |  | 45 | 12 | 7 | 0,156 |  |
|  |  |  |  |  |  | 41 | 24 | 9 | 0,219 |  |
|  | No Augmentation |  |  |  |  | 46 | 1 | 22 | 0,478 |  |
|  |  |  |  |  |  | 46 | 6 | 27 | 0,587 |  |
|  |  |  |  |  |  | 45 | 12 | 25 | 0,556 |  |
|  |  |  |  |  |  | 37 | 24 | 28 | 0,757 |  |
| Iannotti, 2006 [62] | Overall | O | L, MS |  | MRI, US | 30 | 14 | 12 | 0,4 |  |
|  | Control |  |  |  |  | 15 | 14 | 6 |  |  |
|  | Augmentation |  |  |  |  | 15 | 14 | 11 |  |  |
| Jenssen, 2018 [65] | Overall | A | S, M | SR | MRI | 118 | 3 | 10 | 0.085 |  |
|  |  |  |  |  |  | 113 | 12 | 12 | 0.106 |  |
|  | 3w immobilization |  |  |  |  | 59 | 3 | 6 | 0.102 | ^a^0_48, 1_12, 2_0, 3_0, 4_0 |
|  |  |  |  |  |  | 56 | 12 | 6 | 0.107 |  |
|  | 6w immobilization |  |  |  |  | 59 | 3 | 4 | 0.068 | 0_48, 1_12, 2_0, 3_0, 4_0 |
|  |  |  |  |  |  | 57 | 12 | 6 | 0.105 |  |
| Koh, 2014 [67] | Overall | A | M, L | SR | MRI | 88 | 6.8 | 9 | 0.102 | ^b^GFDI 1.1 |
|  | 4w immobilization |  |  |  |  | 40 | 6.8 | 5 | 0.125 | GFDI 1.2 |
|  | 8w immobilization |  |  |  |  | 48 | 6.8 | 4 | 0.083 | GFDI 1.1 |
| Mazzocca, 2017 [69] | Overall | A | S, M, L | SB | MRI | 58 | 6 | 20 | 0.345 | ^a^0_18, 1_32, 2_5, 3_2, 4_0 |
|  | Delayed |  |  |  |  | 27 | 6 | 9 | 0.333 | 0_8, 1_13, 2_4, 3_1, 4_0 |
|  | Early |  |  |  |  | 31 | 6 | 11 | 0.355 | 0_10, 1_19, 2_1, 3_1, 4_0 |
| Sheps, 2019 [70] | Overall | A | S, M, L, MS | SR, DR, SB, TR | US | 165 | 12 | 52 | 0.315 |  |
|  | Early Mobilization |  |  |  |  | 79 | 12 | 24 | 0.304 |  |
|  | Standard Rehabilitation |  |  |  |  | 86 | 12 | 28 | 0.326 |  |
| Lee, 2016 [71] | Overall | A | S, M | SR, SB | MRI | 128 | 12 | 20 | 0.156 |  |
|  | LHBT tenotomy |  |  |  |  | 56 | 12 | 9 | 0.161 |  |
|  | LHBT tenodesis |  |  |  |  | 72 | 12 | 11 | 0.153 |  |
| Nam, 2018 [74] | Overall | A | S, M, L, MS | SR | MRI | 41 | 12 | 6 | 0.146 |  |
|  | Limited Bursectomy |  |  |  |  | 20 | 12 | 3 | 0.15 |  |
|  | Extensive Bursectomy |  |  |  |  | 21 | 12 | 3 | 0.143 |  |
| Osti, 2013 [76] | Overall | A | M, L | SR | MRI | 57 | 29 | 5 | 0.088 |  |
|  | Repair and microfractures |  |  |  |  | 28 | 29 | 2 | 0.071 |  |
|  | Repair only |  |  |  |  | 29 | 29 | 3 | 0.103 |  |
| Liu, 2017 [15] | Overall | A, MO |  | SR, DR | US | 99 | 12 | 9 | 0.091 |  |
|  | Arthroscopic |  |  |  |  | 50 | 12 | 5 | 0.1 |  |
|  | Mini open |  |  |  |  | 49 | 12 | 4 | 0.082 |  |
| van der Zwaal, 2013 [79] | Overall | A, MO | S, M | SB | US | 95 | 12 | 14 | 0.147 | ^a^0_38, 1_42, 2_15, 3_0, 4_0 |
|  | Arthroscopic |  |  |  |  | 47 | 12 | 8 | 0.17 | 0_17, 1_21, 2_9, 3_0, 4_0 |
|  | Mini open |  |  |  |  | 48 | 12 | 6 | 0.125 | 0_21, 1_21, 2_6, 3_0, 4_0 |
| Rhee, 2012 [80] | Overall | A | M | SB | MRI | 110 | 6.75 | 14 | 0.127 |  |
|  | Knotless |  |  |  |  | 51 | 6.4 | 3 | 0.059 | ^b^GFDI 6.4 |
|  | Knot-Tying |  |  |  |  | 59 | 7.1 | 11 | 0.186 | GFDI 7.1 |
| Keener, 2014 [81] | Overall | A | S, M | DR, SB | US | 116 | 12 | 9 | 0.078 |  |
|  | Standard Rehabilitation |  |  |  |  | 63 | 12 | 6 | 0.095 |  |
|  | Immobilization |  |  |  |  | 53 | 12 | 3 | 0.057 |  |

**Abbreviation** *RC:* Rotator Cuff, *PRP:* Platelet-rich plasma, *A:* Arthroscopy, *O:* Open, *MO:* Mini-open, *S:* Small, *M:* Medium, *L:* Large, *MS:* Massive, *SR:* Single-row, *DR:* Double-row, *SB:* Suture bridge, *TR:* Transosseous, *MRI:* Magnetic Resonance Imaging, *US:* Ultrasound, *CTA:* Computed Tomographic Arthrography, *SSP:* Supraspinatus, *ISP:* Infraspinatus, *SSC:* Subscapularis

^a^ According to Goutallier classification, for each muscle the grade of fatty infiltration and number of patients are reported as grade_number of patients.

^b^ *GFDI* Global Fatty Degeneration Index
