# Supplementary material for: Retear rates after rotator cuff surgery: a systematic review and meta-analysis
Source: BMC Musculoskelet Disord. 2021 Aug 31;22:749. doi: 10.1186/s12891-021-04634-6 (PMC8408924; doi:10.1186/s12891-021-04634-6)
Supplement: Supplementary file 3 — Additional file 3. Risk of bias. [file 12891_2021_4634_MOESM3_ESM.docx]

**Additional file 3**

| **Author** | Was the method of randomization adequate? | Was the treatment allocation concealed? | Were the groups similar at baseline regarding the most important prognostic indicators? | Was the patient blinded to the intervention? | Was the personnel blinded to the intervention? | Was the outcomes assessor blinded to the interventions? | Incomplete data addressed? (rehabilitation protocol) | Incomplete data addressed? (tear size) | Incomplete data addressed? (imaging) | Incomplete data addressed? (retears) | Incomplete data addressed? (fatty infiltration) | Was the dropout rate described and accettable? | Was the timing of the outcome assessment in all groups similar? | Were the coiterventions avoided or similar? | Free of selective reporting? | **TOT score** |
| --- | --- | --- | --- | --- | --- | --- | --- | --- | --- | --- | --- | --- | --- | --- | --- | --- |
| Burks, 2009 [33] | 0 | 0 | 0 | 1 | 1 | 0 | 0 | 0 | 0 | 0 | 1 | 0 | 0 | 0 | 0 | 3 |
| Carbonel, 2012 [34] | 0 | 0 | 0 | 1 | 1 | 1 | 0 | 0 | 0 | 0 | 1 | 1 | 0 | 0 | 0 | 5 |
| Castricini, 2010 [35] | 0 | 0 | 0 | 2 | 0 | 0 | 0 | 0 | 0 | 0 | 1 | 0 | 0 | 0 | 0 | 3 |
| D'Ambrosi, 2016 [36] | 0 | 0 | 0 | 2 | 0 | 0 | 0 | 0 | 0 | 0 | 1 | 0 | 0 | 0 | 0 | 3 |
| Ebert, 2017 [37] | 0 | 2 | 0 | 2 | 0 | 2 | 1 | 0 | 0 | 0 | 1 | 0 | 0 | 2 | 0 | 10 |
| Flury,2016 [38] | 0 | 0 | 0 | 0 | 0 | 0 | 2 | 0 | 0 | 0 | 2 | 0 | 0 | 1 | 2 | 7 |
| Franceschi,2007 [39] | 0 | 0 | 0 | 1 | 1 | 1 | 1 | 0 | 0 | 0 | 1 | 2 | 0 | 0 | 0 | 7 |
| Franceschi, 2016 [40] | 0 | 0 | 0 | 1 | 1 | 1 | 0 | 0 | 0 | 0 | 1 | 2 | 0 | 1 | 0 | 7 |
| Gartsman, 2013 [12] | 0 | 2 | 0 | 1 | 1 | 1 | 0 | 2 | 0 | 0 | 1 | 2 | 0 | 0 | 2 | 12 |
| Gumina, 2012 [41] | 0 | 0 | 0 | 0 | 2 | 2 | 0 | 0 | 0 | 0 | 2 | 2 | 0 | 0 | 0 | 8 |
| Gumina, 2012 [42] | 0 | 2 | 0 | 1 | 1 | 1 | 0 | 0 | 0 | 0 | 0 | 2 | 0 | 0 | 0 | 7 |
| Jacquot, 2014 [43] | 2 | 2 | 0 | 0 | 0 | 2 | 2 | 0 | 0 | 0 | 1 | 0 | 0 | 0 | 0 | 9 |
| Jo,2013 [44] | 0 | 0 | 0 | 0 | 0 | 0 | 0 | 0 | 0 | 0 | 0 | 0 | 0 | 0 | 0 | 0 |
| Kim,2016 [45] | 0 | 0 | 0 | 0 | 0 | 0 | 2 | 0 | 0 | 0 | 0 | 2 | 0 | 0 | 0 | 4 |
| Koh,2011 [28] | 0 | 0 | 0 | 1 | 0 | 0 | 0 | 0 | 0 | 0 | 0 | 2 | 0 | 0 | 0 | 3 |
| Lapner,2012 [13] | 0 | 0 | 0 | 0 | 0 | 0 | 0 | 0 | 0 | 0 | 1 | 0 | 0 | 0 | 0 | 1 |
| Ma,2012 [46] | 0 | 0 | 0 | 2 | 0 | 2 | 0 | 0 | 0 | 0 | 1 | 0 | 0 | 0 | 0 | 5 |
| Malavolta,2018 [47] | 0 | 0 | 0 | 0 | 0 | 0 | 0 | 0 | 0 | 0 | 1 | 0 | 0 | 0 | 0 | 1 |
| Pandey,2016 [48] | 0 | 0 | 0 | 0 | 0 | 0 | 0 | 0 | 0 | 0 | 1 | 0 | 0 | 0 | 0 | 1 |
| Randelli,2017 [49] | 0 | 0 | 0 | 0 | 0 | 0 | 0 | 0 | 0 | 0 | 1 | 0 | 0 | 0 | 0 | 1 |
| Randelli,2011 [50] | 0 | 0 | 0 | 0 | 0 | 0 | 0 | 0 | 0 | 0 | 1 | 0 | 0 | 0 | 0 | 1 |
| Rodeo,2012 [51] | 0 | 2 | 0 | 0 | 0 | 0 | 0 | 0 | 0 | 0 | 1 | 0 | 0 | 0 | 0 | 3 |
| Ruiz-moneo,2013 [52] | 0 | 0 | 0 | 0 | 0 | 0 | 2 | 0 | 2 | 2 | 1 | 0 | 0 | 2 | 0 | 9 |
| Shin, 2012 [53] | 2 | 2 | 0 | 2 | 2 | 2 | 0 | 0 | 0 | 0 | 2 | 2 | 0 | 0 | 0 | 14 |
| Walsh,2018 [54] | 0 | 0 | 0 | 0 | 0 | 0 | 0 | 0 | 0 | 0 | 1 | 0 | 0 | 0 | 0 | 1 |
| Wang, 2015 [55] | 0 | 2 | 0 | 2 | 2 | 0 | 0 | 0 | 0 | 0 | 1 | 0 | 0 | 0 | 0 | 7 |
| Weber, 2012 [56] | 2 | 2 | 0 | 0 | 0 | 0 | 2 | 0 | 0 | 0 | 1 | 0 | 0 | 0 | 0 | 7 |
| Zumstein, 2016 [57] | 0 | 0 | 0 | 0 | 0 | 0 | 0 | 2 | 0 | 0 | 0 | 2 | 0 | 0 | 0 | 4 |
| Barber, 2012 [58] | 0 | 0 | 0 | 2 | 0 | 0 | 0 | 0 | 0 | 0 | 1 | 2 | 0 | 0 | 0 | 5 |
| Cai, 2018 [59] | 0 | 0 | 0 | 2 | 2 | 0 | 0 | 0 | 0 | 0 | 1 | 0 | 0 | 0 | 0 | 5 |
| Greiner, 2015 [60] | 0 | 0 | 0 | 2 | 2 | 0 | 0 | 2 | 0 | 0 | 2 | 2 | 0 | 0 | 0 | 10 |
| Avanzi, 2019 [87] | 0 | 0 | 0 | 0 | 0 | 0 | 2 | 0 | 0 | 0 | 1 | 0 | 0 | 0 | 0 | 3 |
| Lamas, 2019 [61] | 0 | 0 | 0 | 2 | 0 | 0 | 2 | 0 | 0 | 0 | 1 | 2 | 0 | 0 | 0 | 7 |
| Iannotti, 2006 [62] | 0 | 0 | 0 | 2 | 0 | 0 | 0 | 0 | 0 | 2 | 1 | 0 | 0 | 0 | 0 | 5 |
| Ide, 2017 [63] | 0 | 0 | 0 | 2 | 2 | 0 | 0 | 0 | 0 | 0 | 0 | 2 | 0 | 0 | 0 | 6 |
| Cuff, 2012 [64] | 0 | 0 | 0 | 2 | 2 | 0 | 0 | 2 | 0 | 0 | 1 | 0 | 0 | 2 | 0 | 9 |
| Jenssen, 2018 [65] | 0 | 0 | 0 | 0 | 0 | 0 | 0 | 0 | 0 | 0 | 0 | 0 | 0 | 0 | 0 | 0 |
| Kim, 2012 [66] | 0 | 0 | 0 | 2 | 2 | 2 | 0 | 0 | 0 | 0 | 1 | 0 | 0 | 0 | 0 | 7 |
| Koh,2014 [67] | 0 | 0 | 0 | 2 | 0 | 0 | 0 | 0 | 0 | 0 | 0 | 0 | 0 | 0 | 0 | 2 |
| Lee, 2012 [68] | 2 | 2 | 0 | 2 | 0 | 0 | 0 | 0 | 0 | 0 | 0 | 0 | 0 | 0 | 0 | 6 |
| Mazzocca, 2017 [69] | 0 | 0 | 0 | 0 | 0 | 0 | 0 | 0 | 0 | 0 | 0 | 0 | 0 | 0 | 0 | 0 |
| Sheps, 2019 [70] | 0 | 0 | 0 | 2 | 0 | 0 | 2 | 0 | 0 | 0 | 1 | 0 | 0 | 0 | 0 | 5 |
| Lee, 2016 [71] | 0 | 0 | 0 | 0 | 0 | 0 | 2 | 0 | 0 | 0 | 1 | 0 | 0 | 0 | 0 | 3 |
| Carr, 2017 [72] | 0 | 2 | 0 | 2 | 2 | 0 | 2 | 1 | 0 | 0 | 1 | 2 | 0 | 2 | 0 | 14 |
| Bidwai, 2015 [73] | 0 | 1 | 0 | 1 | 1 | 0 | 2 | 0 | 2 | 2 | 1 | 0 | 1 | 0 | 0 | 11 |
| Nam, 2018 [74] | 0 | 0 | 0 | 0 | 0 | 0 | 2 | 0 | 0 | 0 | 1 | 0 | 0 | 0 | 0 | 3 |
| Boehm, 2005 [75] | 0 | 0 | 0 | 2 | 0 | 2 | 2 | 0 | 0 | 0 | 1 | 0 | 0 | 0 | 0 | 7 |
| Osti, 2013 [76] | 0 | 0 | 0 | 2 | 0 | 0 | 0 | 2 | 0 | 0 | 1 | 0 | 0 | 0 | 0 | 5 |
| Tirefort, 2019 [77] | 0 | 0 | 0 | 2 | 2 | 0 | 0 | 0 | 0 | 0 | 2 | 0 | 0 | 0 | 0 | 6 |
| Liu, 2017 [15] | 0 | 0 | 0 | 0 | 0 | 0 | 0 | 2 | 0 | 0 | 1 | 0 | 0 | 0 | 0 | 3 |
| Zhang, 2016 [78] | 1 | 1 | 0 | 1 | 1 | 1 | 1 | 0 | 0 | 0 | 0 | 1 | 0 | 0 | 0 | 7 |
| van der Zwaal, 2013 [79] | 0 | 0 | 0 | 0 | 0 | 0 | 0 | 0 | 0 | 0 | 0 | 0 | 0 | 0 | 0 | 0 |
| Rhee, 2012 [80] | 0 | 0 | 0 | 2 | 2 | 0 | 0 | 1 | 0 | 0 | 0 | 0 | 0 | 0 | 0 | 5 |
| Keener, 2014 [81] | 0 | 0 | 0 | 2 | 0 | 0 | 0 | 0 | 0 | 0 | 1 | 0 | 2 | 0 | 0 | 5 |
| Antuña, 2013 [82] | 0 | 0 | 0 | 2 | 2 | 0 | 2 | 0 | 0 | 0 | 1 | 0 | 0 | 0 | 0 | 7 |
| Kim, 2012 [83] | 2 | 2 | 0 | 1 | 1 | 1 | 0 | 0 | 0 | 0 | 0 | 2 | 0 | 0 | 0 | 9 |
| De Roo, 2015 [84] | 2 | 2 | 0 | 2 | 2 | 0 | 0 | 0 | 0 | 0 | 1 | 1 | 0 | 2 | 0 | 12 |
| Lamber Heerspink, 2015 [85] | 0 | 0 | 0 | 2 | 2 | 2 | 0 | 2 | 0 | 0 | 1 | 0 | 0 | 2 | 0 | 11 |
| Moosmayer, 2019 [86] | 0 | 0 | 0 | 2 | 2 | 0 | 0 | 0 | 2 | 2 | 1 | 2 | 0 | 0 | 0 | 11 |
